# Unveiling the Osteogenic Potential of Tetracyclines: A Comparative Study in Human Mesenchymal Stem Cells

**DOI:** 10.3390/cells12182244

**Published:** 2023-09-10

**Authors:** Victor Martin, Ana Francisca Bettencourt, Catarina Santos, Maria Helena Fernandes, Pedro Sousa Gomes

**Affiliations:** 1 BoneLab—Laboratory for Bone Metabolism and Regeneration, Faculty of Dental Medicine, University of Porto, 4200-393 Porto, Portugal; vmartin@fmd.up.pt (V.M.); mhfernandes@fmd.up.pt (M.H.F.); 2 LAQV/REQUIMTE, University of Porto, 4050-453 Porto, Portugal; 3 Research Institute for Medicines (iMed.ULisboa), Faculdade de Farmácia, Universidade de Lisboa, 1649-003 Lisboa, Portugal; asimao@ff.ulisboa.pt; 4 CQE Instituto Superior Técnico, Universidade de Lisboa, 1049-001 Lisboa, Portugal; catarina.santos@estsetubal.ips.pt; 5 EST Setúbal, CDP2T, Instituto Politécnico de Setúbal, 2910-761 Setúbal, Portugal

**Keywords:** tetracyclines, mesenchymal stem cells, osteogenesis, drug repurposing, Wnt signaling, Hedgehog signaling

## Abstract

Tetracyclines (TCs) are a class of broad-spectrum antibiotics with diverse pharmacotherapeutic properties due to their various functional groups being attached to a common core structure. Beyond their antibacterial activity, TCs trigger pleiotropic effects on eukaryotic cells, including anti-inflammatory and potentially osteogenic capabilities. Consequently, TCs hold promise for repurposing in various clinical applications, including bone-related conditions. This study presents the first comprehensive comparison of the in vitro osteogenic potential of four TCs—tetracycline, doxycycline, minocycline, and sarecycline, within human mesenchymal stem cells. Cultures were characterized for metabolic activity, cell morphology and cytoskeleton organization, osteogenic gene expression, alkaline phosphatase (ALP) activity, and the activation of relevant signaling pathways. TCs stimulated actin remodeling processes, inducing morphological shifts consistent with osteogenic differentiation. Osteogenic gene expression and ALP activity supported the osteoinduction by TCs, demonstrating significant increases in ALP levels and the upregulation of RUNX2, SP7, and SPARC genes. Minocycline and sarecycline exhibited the most potent osteogenic induction, comparable to conventional osteogenic inducers. Signaling pathway analysis revealed that tetracycline and doxycycline activate the Wnt pathway, while minocycline and sarecycline upregulated Hedgehog signaling. Overall, the present findings suggest that TCs promote osteogenic differentiation through distinct pathways, making them promising candidates for targeted therapy in specific bone-related disorders.

## 1. Introduction

Tetracyclines (TCs) are a class of broad-spectrum bacteriostatic antibiotics characterized by a four-ring carbocyclic structure. Chemically, TCs differ from one another due to the varying functional groups attached to the core structure [1,2]. The therapeutic effectiveness of TCs has been extensively demonstrated in the management of various infectious conditions caused by Gram-positive and Gram-negative bacteria, rickettsia, and mycoplasma, comprehensively reviewed in previous studies [3,4]. The primary antibacterial mechanism of action for TCs relies on their ability to bind to multiple target sites within the bacterial ribosomal structure. This binding interferes with tRNA binding and disrupts protein synthesis, resulting in the inhibition of cell growth in a bacteriostatic way [5]. Additional mechanisms, relying for instance on membrane perturbation, have been described [5]. These findings provide further evidence of the chemical and biological versatility of TCs, as they can adapt and modify their conformation in an environment-dependent manner [6], further highlighting the dynamic nature of TCs and their ability to exert multiple effects on bacterial cells [2]. 

In addition to their well-established chemotherapeutic effectiveness, TCs have been associated with distinct pleiotropic effects that outreach into eukaryotic cells and tissues [2]. These effects include anti-inflammatory, anti-metalloproteinase, antitumor, neuroprotective, antioxidant, and cation chelation activities [7]. As a result, TCs have been investigated and tested for a range of diseases, such as osteoporosis, neuropathies, acne, rheumatoid arthritis, periodontitis, certain types of carcinomas, and recently, COVID-19, endorsing its repurposing by the pharmaceutical industry [7,8,9,10,11,12,13]. In this frame, TCs have garnered attention for bone-tissue-related applications due to their high affinity and remarkable retention in the bone matrix, as well as their ability to modulate the functionality of bone cells [14,15]. This realization has prompted the development of distinctive drug-carrier platforms (e.g., nanoparticles, microparticles, cements, 3D printing scaffolds, coatings, and fibers), which have been conjugated with TCs using various approaches [16,17]. The goal of these carrier systems was to enable the targeted release of TCs to the bone, capitalizing on their antibacterial and/or non-antibacterial effects in situ [18,19]. 

TCs possess the ability to chelate metallic ions, including calcium, resulting in a high affinity for binding to the hydroxyapatite-rich inorganic matrix of the bone tissue [20]. In addition to their bone-targeting capability, TCs also appear to modulate the bone formation and bone resorption processes [1,21]. They seem to contribute to the inhibition of bone resorption by blocking the metal-dependent matrix metalloproteinases (MMPs)—namely gelatinase and collagenases [22]. Furthermore, TCs were shown to inhibit osteoclastogenesis by inducing the apoptosis of osteoclasts [23], and by blocking the NFATc1 signaling pathway upon RANK-RANKL signaling modulation, as demonstrated in vitro and in vivo [1,24,25]. Preliminary evidence suggests that TCs may also induce the osteogenic program [21,26,27]. However, the specific signaling pathways through which TCs modulate osteogenic commitment in physiological conditions have not been previously investigated. Furthermore, although some individual TC molecules have been reported to have potential bone-modulatory effects, there is currently a lack of comprehensive comparative assessments between different drugs. 

The primary objective of this study was to assess the in vitro osteogenic potential of four TCs, representing all three generations, to understand their ability to influence the osteogenic commitment of human mesenchymal stem cells. The tested TCs were tetracycline (TETRA, 1st gen.), doxycycline (DOXY 2nd, gen.), minocycline (MINO, 2nd gen.), and sarecycline (SARE 3rd gen.), assayed at different concentrations. The evaluation of the biological response encompassed a comprehensive analysis of various aspects in the established cell cultures. These included evaluating the metabolic activity, observing cell morphology and organization, determining the expression and activity of osteogenic markers, and investigating the activation of relevant signaling pathways. 

## 2. Materials and Methods

The response to different concentrations (0.1, 1.0, and 10 µg/mL) of selected tetracyclines (TETRA, DOXY, MINO, and SARE) (Table 1) was evaluated in vitro, using precursor osteoblastic populations–human bone marrow stem cells (HBMSCs, Lonza, Catalog #: PT-2501). Before the biological evaluation, HBMSCs were characterized by flow cytometry and found to be positive for CD105, CD73, and CD90, and negative for CD45, CD34, and CD31 markers. Cells from the 4th passage were used in this study. The HBMSCs were expanded in α-Minimal Essential Medium (α-MEM) culture medium, supplemented with 10% fetal bovine serum (FBS), 100 IU/mL penicillin, 100 μg/mL streptomycin, and 2.5 µg/mL amphotericin B (all from Gibco, Gaithersburg, MD, USA). The cultures were maintained at 37 °C with 5% CO_2_ in air. Once the cultures reached approximately 70% confluence, they were detached and sub-cultured at a density of 10,000 cells/cm^2^ in the defined culture medium. After 24 h, the culture medium was removed, and the cells were incubated with a complete culture medium supplemented with 10% PBS-antibiotic solution, containing the designated concentrations of the tetracyclines, for up to 21 days. Control cultures were grown in basal culture medium supplemented with 10% PBS without any tetracyclines (C-BAS), while osteogenic-induced cultures were grown in 10 mM β-glycerophosphate, 50 µg/mL ascorbic acid and 10 nM dexamethasone (all from Sigma-Aldrich, St. Louis, MO, USA). The culture medium was changed twice a week. 

### 2.1. Metabolic Activity Assay

The metabolic activity of the cell cultures was assessed using the tetrazolium dye (MTT). At selected periods, a 10% MTT solution (5 mg/mL, Sigma-Aldrich, St. Louis, MO, USA) was added to cultured cells and incubated for 3 h. Afterwards, the supernatant was removed, and the formed crystals were dissolved with dimethyl sulfoxide (DMSO). The absorbance of the resulting-colored solution was measured at 550 nm using a microplate reader (Synergy HT; BioTek, Winooski, VT, USA). The obtained data were normalized to the values of the control group (C-BAS), set as 1.0. The assay was performed in quintuplicates, across three independent experiments.

### 2.2. Cell Morphology

Cell morphology was assessed using fluorescence microscopy upon F-actin and mitochondria staining, using phalloidin-conjugated Alexa Fluor 488 (Molecular Probes, Eugene, OR, USA) and MitoSpy™ Red FM (Biolegend, San Diego, CA, USA), respectively. Nuclei were counterstained with Hoechst 33342 (Enzo, Farmingdale, NY, USA). Stained cells were visualized using a Celena S digital imaging system (Logos Biosystems, Annandale, VA, USA). To perform the staining, at selected time points, the cultures were incubated with MitoSpy™ Red FM for 30 min at 37 °C. Subsequently, the cells were fixed with 3.7% paraformaldehyde, permeabilized with 0.1% Triton-X and blocked with bovine serum albumin (Sigma-Aldrich, St. Louis, MO, USA) to prevent non-specific interactions, at room temperature. The cells were then stained with phalloidin-conjugated Alexa Fluor 488 and Hoechst 33342, for F-actin and nucleus staining, respectively. Stained cells were subjected to microscopic analysis. Images were treated using the ImageJ software v.1.53k (National Institutes of Health, Bethesda, MD, USA). The assay was conducted in triplicates, across three independent experiments.

### 2.3. Assessment of Gene Expression by Quantitative PCR (qPCR)

Total RNA was isolated from cell cultures, at selected timepoint, with Trizol reagent (Invitrogen, Carlsbad, CA, USA) following the manufacturer’s protocol. RNA concentration and quality were assessed with an absorbance reading (260/280 nm) using a Take3 module (Gen5, BioTek, Winooski, VT, USA) and a microplate reader (Synergy HT; BioTek), and samples with A260/A280 ratio of approx. 2.0 proceeded to cDNA conversion. The conversion to cDNA was achieved using a two-step reverse transcription quantitative NZY Kit (NZYTech, Lisbon, Portugal), normalizing the RNA concentration to 300 ng/µL, followed by the addition of RNAse (NZYTech, Lisbon, Portugal). The qPCR reaction was performed in a CFX384 real-time PCR system (Bio-Rad, Hercules, CA, USA) with the iTaq Universal SYBR green Supermix (Bio-Rad, Hercules, CA, USA), according to the Bio-Rad cycling protocol (activation–2 min at 95 °C; 40 cycles of denaturation/annealing, 5 sec at 95 °C and 30 sec at 60 °C; melt curve, 65–95 °C). Selected primers were purchased from Bio-Rad (Table 2), and the negative control was set using DEPC for each primer. Finally, Bio-Rad CFX Maestro 1.1 (v.4.1.24, Bio-Rad, Hercules, CA, USA) was used to collect data and check the reaction’s quality control, where only samples with amplification efficiency between 90 and 100 were used, with a linear standard curve (R^2^) greater than 0.98. The relative quantification of each target gene was normalized to beta-actin levels (housekeeping gene) and calculated via the 2^–ΔΔCt^ method. Amplification reactions were conducted in quintuplicates. RNA extraction was performed using five independent assays, and each sample was converted to cDNA in duplicates.

### 2.4. Alkaline Phosphatase (ALP) Histochemical Staining

At defined time points, HBMSCs were fixed in 1.5% glutaraldehyde for 10 min. Then, cells were stained with α-naphthyl acid phosphate (2 mg/mL) and Fast Blue R (2 mg/mL) solutions and incubated for 1 h protected from the light. The presence of ALP was noticed by brown-to-black staining according to the relative amount of the enzyme. Images were taken using a Zeiss Axiolab 5 microscope coupled with an Axiocam 5 Color Camera. The percentage of the ALP-stained area of obtained images was calculated using ImageJ software (v.1.53k, National Institutes of Health, Bethesda, MD, USA) using the Otsu algorithm to set the threshold. The assay was conducted in triplicates, across three independent experiments.

### 2.5. Statistical Analysis

Statistical analysis was conducted using the SPSS software (IBM, v.27). The normality of the datasets was assessed using the Shapiro–Wilk test. For datasets that followed a normal distribution, one-way ANOVA was performed, followed by Tukey’s multiple comparisons test. Non-parametric datasets were analyzed using the Kruskal–Wallis test, followed by Dunn’s multiple comparison tests. The significance level was set at *p* ≤ 0.05. The data were presented as mean ± standard deviation (SD) to provide a measure of the central tendency and variability of the results.

## 3. Results

**Figure 1:**
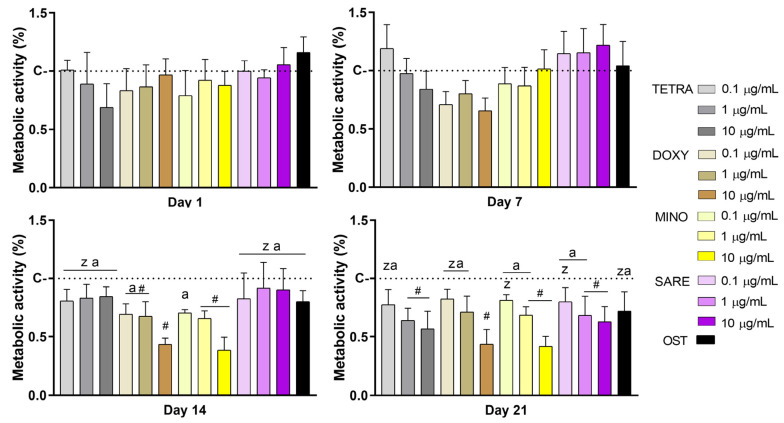
Metabolic activity/cell viability of HBMSC cultures incubated with tetracyclines at different concentrations—MTT assay. TETRA corresponds to tetracycline; DOXY to doxycycline; MINO to minocycline; SARE to sarecycline; and OST to osteogenic-induced cultures. The color scale corresponds to the tetracycline/drug and concentrations of 0.1, 1.0, and 10 µg/mL. Experimental group values were normalized by the basal control (C) for each timepoint, set as 1.0. (#) different from C; (z) different from DOXY at 10 µg/mL, (a) different from MINO at 10 µg/mL. (n = 5, *p* ≤ 0.05).

Throughout the culture period, both the basal control and the experimental conditions exhibit a gradual increase in the metabolic activity/cell viability, from day 1 to day 21. When a comparative assessment was conducted for each time point (Figure 1), regarding days 1 and 7, no significant differences were observed between conditions, regardless of the tested TCs or concentrations. However, a tendency for decreased values could be seen in the TETRA group at a concentration of 10 µg/mL, at day 1; while the DOXY group presented this trend across all concentrations at day 7. Moreover, TETRA at 0.1 µg/mL and the SARE group at all concentrations showed a trend for increased MTT reduction values, compared to the basal control (C-BAS), also at day 7. No significant differences were found regarding the comparison to the osteogenic-induced condition (OST) at early time points. At day 14, significant differences were observed, with the MINO and DOXY groups showing significantly lower metabolic activity, particularly at a concentration of 10 µg/mL. In contrast, the TETRA and SARE groups exhibited similar metabolic activity to the basal control (C-BAS). The OST condition showed a trend for reduced metabolic activity values. Finally, at day 21, all experimental groups had MTT reduction levels lower than those of the basal control, with significant differences observed at concentrations of 1 and 10 µg/mL for all TCs, except for DOXY at 1 µg/mL. The OST cultures maintained the trend of reduced metabolic activity values. Lower concentrations (0.1 µg/mL) of all TCs showed no significant differences compared to the control.

**Figure 2:**
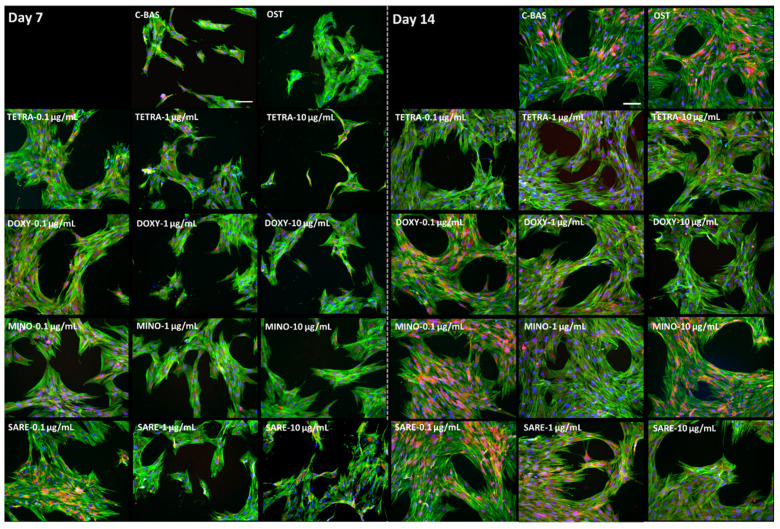
Representative fluorescent images of cellular morphology of HBMSC cultures at day 7 and day 14, incubated with tetracyclines at different concentrations. The cellular actin cytoskeleton was stained in green; nuclei were counter-stained in blue; and mitochondria were dyed in red. The scale bar corresponds to 100 μm. TETRA corresponds to tetracycline; DOXY to doxycycline; MINO to minocycline; SARE to sarecycline; C-BAS to basal control; and OST to osteogenic-induced cultures.

HBMSCs’ morphology was observed and documented after 7 and 14 days of culture (Figure 2). On day 7, the control group (C-BAS) exhibited a dispersed distribution of cells with a uniform elongated morphology. Actin stress fibers extended across the scattered cytoplasm in a parallel fashion, accompanied by filopodial projections indicating an attempt to establish direct contact with nearby cells. Nuclei were prominently stained, while mitochondrial staining was faint and concentrated at the perinuclear region. Osteogenic-induced cells (OST) presented a similar pattern of culture organization, although some cells exhibited a more polygonal shape. Cultures exposed to tetracyclines, at different concentrations, displayed a comparable culture organization and morphological pattern. By day 14, an increased cellular density was observed in all conditions, although there were some variations in the overall cell number. Cells were arranged in a trabecular-like organization, and there was a more intense reddish perinuclear accent due to stronger mitochondrial staining. Compared to the control (C-BAS), the TCs-exposed conditions and OST showed rearrangement of the actin cytoskeleton, with thicker actin filament bundles observed at the cell periphery.

**Figure 3:**
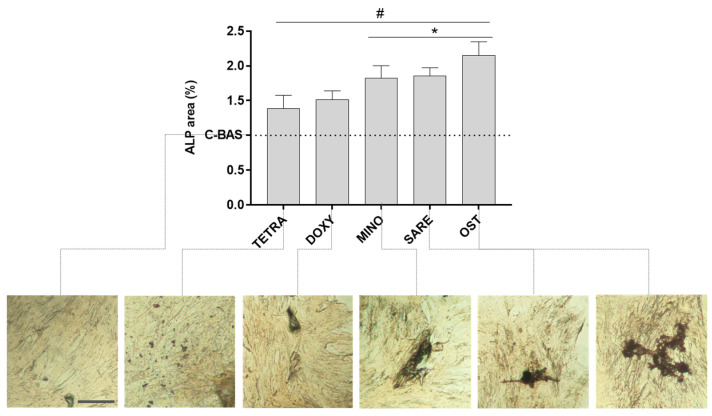
Osteogenic activity of HBMSCs cultured with TCs at 1.0 µg/mL. Quantitative analysis and representative histochemical staining of ALP activity displayed as a normalized percentage as compared to the basal control (C-BAS) set as 1.0. TETRA corresponds to tetracycline; DOXY to doxycycline; MINO to minocycline; SARE to sarecycline; C-BAS to basal control; and OST to osteogenic-induced cultures. The scale bar corresponds to 100 μm. (#) different from control; (*) different from TETRA and DOXY. n = 5, *p* ≤ 0.05.

**Figure 4:**
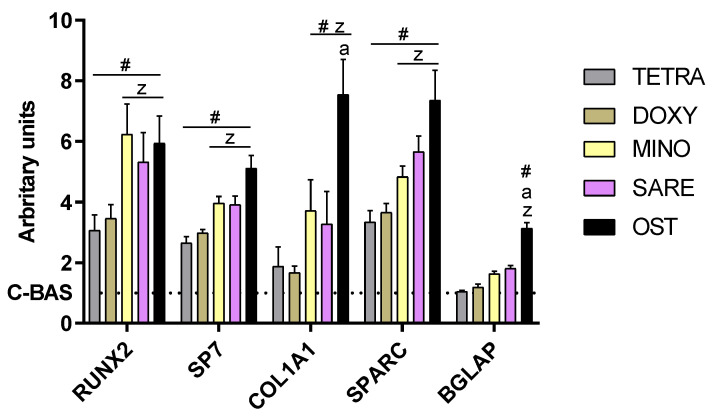
Relative gene expression of osteogenesis-related genes using quantitative PCR assessment. Values were normalized via the 2^–ΔΔCt^ method. TETRA corresponds to tetracycline; DOXY to doxycycline; MINO to minocycline; SARE to sarecycline; C-BAS to basal control; and OST to osteogenic-induced cultures. (#) different from control; (z) different from TETRA and DOXY; (a) different from MINO and SARE. n = 5, *p* ≤ 0.05.

To evaluate and compare the ability of TCs to modulate the osteogenic program in precursor populations, 14-day HBMSCs cultures incubated with TCs at 1.0 µg/mL were assessed for alkaline phosphatase (ALP) activity (Figure 3) and osteogenic gene expression (Figure 4). In terms of the ALP histochemical staining, the control cultures (C-BAS) displayed a light-brownish accent, indicating baseline ALP activity. In contrast, the osteogenic-induced cultures (OST) exhibited significantly increased dark staining, indicating a higher level of ALP activity. The experimental groups treated with TCs exhibited an intensified color accent and the presence of focalized areas of darker staining, demonstrating regions of increased enzyme activity compared to the control (C-BAS). This observation was confirmed by the quantitative analysis, where all experimental groups showed significantly higher levels of ALP compared to the control (C-BAS), with MINO and SARE reaching levels like those observed in the OST group. 

In Figure 4, significant differences can be observed among TC- exposed cultures in terms of osteogenic gene expression. The genes *RUNX2*, *SP7*, *COL1A1*, and *SPARC* were significantly upregulated in all experimental groups in comparison to the control (C-BAS). Among these, MINO and SARE groups that induced the highest overall upregulation, to levels like those of OST with *RUNX2* and *SP7*.

**Figure 5:**
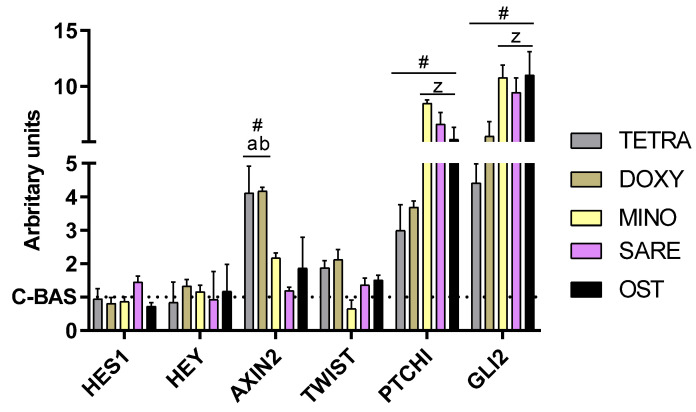
Relative gene expression of relevant osteogenic pathways of cultures using qPCR technique. Values were normalized via the 2^–ΔΔCt^ method. (#) different from control; (z) different from TETRA and DOXY; (a) different from MINO and SARE; (b) different from OST. n = 5, *p* ≤ 0.05.

Furthermore, the potential modulation of relevant signaling pathways was evaluated via the assessment of target genes: *HES1* and *HEY* for the Notch pathway; *AXIN2* and *TWIST* for the Wnt/β-catenin pathway; and *PTCH1* and *GLI2* for the Hedgehog (HH) pathway (Figure 5). No significant differences were reported in *HES1* and *HEY* expression in the presence of TCs or OST. DOXY and TETRA were found to significantly upregulate *AXIN2* expression, further inducing a trend for increased *TWIST* expression. *PTCH1* and *GLI2* expression were upregulated by all TCs, with MINO and SARE inducing the expression to levels like or higher than those attained with OST. 

## 4. Discussion

Tetracyclines (TCs) are broad-spectrum antibacterial agents widely used for the treatment of bacterial infections. Tetracycline, doxycycline, and minocycline are the most frequently used molecules in this class [28,29]. TCs have also demonstrated clinical efficacy in managing dermatological conditions, specifically acne vulgaris and rosacea, with a particular focus on the application of sarecycline [30]. In dermatological and related therapies, both antimicrobial and sub-antimicrobial dosages have proven effective, harnessing the pleiotropic effects of TCs while minimizing potential dose-related adverse side effects [31]. Furthermore, sub-antimicrobial therapies have also been expanded to address other local and systemic conditions, such as periodontal diseases, given their immunomodulatory capabilities [11,32], emphasizing the potential for repurposing the TCs family in various therapeutic areas. 

Tetracyclines (TCs) have shown a remarkable affinity for the bone matrix, potentially attributed to their ability to chelate calcium ions [20]. Consequently, the investigation of TCs’ potential modulatory effects on bone tissue has gained attention. While some studies have explored the potential effects of individual TCs on osteogenic differentiation—such as minocycline, doxycycline [26], and sarecycline [33]—a comparative evaluation within the same experimental settings is still lacking. Additionally, the characterization of the underlying signaling pathways responsible for the observed effects remains unexplored. Therefore, this study aimed to comparatively evaluate the in vitro osteogenic capabilities of the most used TCs (i.e., TETRA, DOXY, MINO, and SARE) at distinct concentrations, representing the three generations of TCs. Furthermore, this research focused on the assessment of relevant signaling pathways’ activation, which may contribute to the modulation of the osteogenic differentiation process. By shedding light on the differential osteogenic potential and underlying signaling mechanisms of these TCs, this research intended to provide valuable insights into their potential therapeutic applications in bone tissue-related conditions.

Human mesenchymal stem cells (HMSCs) were selected as osteoblastic precursors in this study, with consideration given to their origin in the bone marrow. Bone-marrow-derived HMSCs possess an epigenetic memory for osteogenic lineage and exhibit an enhanced capability for osteogenic differentiation compared to cells originating from other tissues [34,35]. To induce osteogenic differentiation as a positive control, cultures were grown in the presence of dexamethasone, ascorbic acid, and β-glycerophosphate, which are well-established in vitro inducers of the osteogenic program [36,37]. Dexamethasone is known to induce and enhance the activity of *RUNX2*, the master transcription factor of the osteoblastic differentiation [36]. Ascorbic acid stimulates collagen type I secretion and subsequent collagen/integrin-mediated signaling, while β-glycerophosphate serves as a source of phosphate for the mineralization of the extracellular matrix, further augmenting the osteogenic signaling [38]. The established cultures were characterized regarding metabolic activity/viability, morphological organization, ALP expression, and gene expression analysis to assess the activation of the osteogenic program and relevant signaling pathways. 

The evaluation of the metabolic activity in the TCs-exposed cultures revealed no significant differences at early time points (days 1 and 7), Figure 1. However, a trend of reduced values was observed at later time points (day 14, for DOXY and MINO groups; and day 21, for all TCs), similarly to the attained in the osteogenic group (OST). Previous studies have indicated that different TCs do not impair the viability/metabolic activity of osteoblastic and precursor populations, particularly at low concentrations [26]. Moreover, TCs have demonstrated cell-specific anti-apoptotic effects [39,40], particularly in mesenchymal-derived populations [41], while also selectively increasing apoptosis in osteoclastic-related populations [23,41]. Additionally, a decrease in metabolic activity is expected in osteogenic differentiating populations, at longer culture timepoints. This reduction can be attributed to the increased differentiation commitment within the osteogenic program, as more differentiated cells have a reduced ability to divide compared to less mature cells [42], in line with the well-established inverse relationship between proliferation and differentiation kinetics [43,44]. It is also essential to consider that higher TCs concentrations may induce cytotoxicity, as evidenced by the significant reduction in the metabolic activity observed with the 10 µg/mL concentration at later time points. Reported TCs’ IC50 values, typically falling within the 3−10 µg/mL range, as observed in different cell populations, suggest a potential negative impact of higher concentrations on cellular behavior [26,45]. 

Despite some variations in the cell number that correlate with differences in the metabolic activity, HBMSCs exposed to TCs displayed a consistent morphology across all groups, resembling the morphology observed in the osteogenic-induced group (OST), Figure 2. This similarity suggests that TCs may contribute to the ongoing osteogenic differentiation of HBMSCs and help to elude senescent and adipogenic profiles, which are typically associated with a rounder cell morphology [46]. Notably, during osteogenic differentiation, cells undergo modifications in their cell shape, adopting a spread and polygonal morphology, and forming thick actin bundles (Figure 2). These alterations are attributable to changes in the actin polymerization status, triggered by the physical and chemical signaling transduction elicited by the osteogenic inducers (i.e., dexamethasone, ascorbic acid, and β-glycerophosphate used within the OST group) [47], and similar to the changes observed in TCs-exposed populations. These findings suggest that TCs may stimulate similar actin remodeling processes as the established osteogenic inducers, further supporting their potential role in promoting the osteogenic differentiation of HBMSCs. 

The activation of the osteogenic program was further validated by assessing ALP activity (Figure 3) and the expression of relevant osteogenic genes (Figure 4), upon exposure to TCs at 1.0 µg/mL. Osteogenic-induced cells (OST) exhibited a significant increase in the ALP activity and expression of key transcription factors, *RUNX2* and *SP7* – coding, respectively, for Runt-related transcription factor 2 and osterix. *RUNX2*, a major transcription factor for osteoblastogenesis, plays a crucial role in regulating the transcription program of lineage-specific genes essential for bone formation through genetic and epigenetic mechanisms [48]. *SP7*, an equally important transcription factor for bone formation and bone matrix deposition, modulates the osteoblastic commitment downstream of *RUNX2* [49]. All TCs demonstrated a significant increase in the activity of ALP and expression of both transcription factors compared to the control group (C-BAS), indicating their potential to enhance osteogenic differentiation. Notably, MINO and SARE groups exhibited ALP activity and gene expression levels like those achieved with OST, reaching 4–6 times higher expression levels than the basal control. Furthermore, MINO and SARE also upregulated the expression of downstream osteogenic genes, including *COL1A1*, which encodes the pro-alpha1 chain of type I collagen, the major protein of the extracellular matrix; and *SPARC*, which encodes osteonectin, a non-collagenous protein with collagen- and hydroxyapatite-binding domains that modulate collagen deposition and influence osteoblastic/osteoclastic differentiation [50]. The expression levels of these genes in the presence of MINO and SARE were like those observed in the OST group, indicating that these TCs effectively promote osteogenic differentiation. Additionally, there was a trend towards the increased expression of *BGLAP*, which encodes osteocalcin—the most abundant non-collagenous protein involved in modulating the mineralization process, and an acknowledged late marker of osteogenic differentiation [51]. This tendency was particularly evident in MINO and SARE groups. Overall, while all TCs were able to induce the osteogenic gene expression program, MINO and SARE exhibited the highest induction levels, comparable to those achieved with OST. These differences in gene expression patterns attained with different TCs may be attributed to their selective ability to activate specific signaling pathways involved in osteogenic differentiation, which is addressed in Figure 5. 

The impact of TCs exposure on Notch signaling was evaluated by the assessment of classical canonical target genes, and, interestingly, neither TCs exposure nor OST induction significantly altered their expression. *HES1* (coding for hairy enhancer of the split-1) and *HEY* (coding for hairy ears, Y-linked), showed no significant changes in their expression levels in response to either OST or TCs treatment. Notch signaling has been reported to play a role in the regulation of osteogenic differentiation, albeit with conflicting findings [52,53]. It has been suggested that Notch signaling may enhance late-stage osteogenesis, particularly during the matrix mineralization process if an inhibitory activity is found at early osteogenic stages by the suppression of the Wnt pathway [52,53]. Given that the assessment of the target genes was conducted on day 14—a transition period between the early and late in vitro osteogenic differentiation—the absence of significant modifications in Notch signaling is apprehensible [53]. 

Regarding the Wnt pathway, the expression of *AXIN2* and *TWIST*—target genes known to regulate the pathways’ activity—was assessed. *AXIN2*, encoding the axis inhibition proteins 2, is an endogenous inhibitor providing negative feedback to regulate the pathways’ signaling [54], whilst *TWIST* encodes the Twist Family BHLH Transcription Factor 1, an early transcription factor able to inhibit chondrogenic development in favor of osteogenesis [55]. The Wnt signal transduction pathway, particularly the canonical pathway initiated by distinct Wnt ligands, plays a crucial role in bone formation and the commitment of precursor populations to osteogenesis [56]. The activation of this pathway involves the stabilization b-catenin by inhibiting its phosphorylation, which leads to cytoplasmic accumulation and subsequent nuclear translocation. Once in the nucleus, b-catenin binds to T-cell factor/lymphoid enhancer factor (TCF/LEF) transcription factors, thereby activating the transcription of downstream genes associated with the osteogenic differentiation [56]. In this study, the exposure to TETRA and DOXY significantly upregulated the expression of *AXIN2*, further inducing a positive trend for *TWIST* expression, substantiating the activation of Wnt signaling during the verified osteogenic induction of HBMSCs. These findings are in line with previous research demonstrating the ability of DOXY to enhance osteogenic commitment via an increased Wnt signaling, both in vitro—in osteogenic-compromised bone marrow mesenchymal stromal cells [57], and in vivo by promoting alveolar bone repair [58]. MINO and SARE were found not to significantly influence the expression of Wnt targets, suggesting that the attained osteogenic induction was majorly attained via a distinct signaling. 

Indeed, MINO and SARE exposure exhibited a notable association with increased Hedgehog (HH) signaling, as supported by the upregulation of the target genes *PTCH1* and *GLI2*. The expression levels of these genes in the MINO and SARE groups were comparable to, or even higher than, those observed in the OST group. In the HH pathway, signaling is initiated by the activation of the Smoothened (SMO) receptor, leading to the activation of GLI transcription factors and subsequent upregulation of target genes, including *PTCH1* (a negative regulator of the pathway) and the GLI transcription factors, such as *GLI2* [59]. Previous research has linked HH signaling to the promotion of the osteogenic differentiation of HMSCs through the increased expression of key transcription factors, including *RUNX2* and *SP7* [60,61]. Specifically, the upregulation of *GLI2* has been correlated with increased RUNX2 activity, as these two factors physically interact and synergistically enhance the osteogenic induction [62]. Moreover, a positive feedback loop has been described, involving the *GLI2* signaling and the induction of the insulin-like growth factor 2 (Igf2), an established inducer of osteogenesis [63]. While evidence on TCs-mediated HH signaling in bone-related cells/tissues is limited, previous studies have associated MINO with elevated HH signaling in various cellular populations, speculating on its potential effectiveness in neuromodulation [64]. 

The differentiated biological outcomes and signal modulation observed with the distinct TCs may find origin in chemical dissimilarities. This divergence could be potentially attributed to differences in their chemical structure, particularly at the critical C7 position of the D ring. Interestingly, both SARE and MINO share modifications in in this very C7 position of the D ring, with the addition of distinct amino derivative groups (Table 1). Alterations in the C7 position are considered a major structural optimization, being constantly exploited in the development of new TC molecules [65,66]. In fact, key functional traits such as antibacterial potency and spectrum and interactions between TCs and bacterial mRNA, as well as calcium chelation rate, can be directly manipulated by the addition of functional groups at the C7 position [66]. This highlights the potential relevance of this specific alteration in shaping the interaction and functional modulation of eukaryotic cells, although further studies are needed to fully uncover the implications of this issue.

## 5. Conclusions

The investigation of four commonly used tetracyclines (i.e., tetracycline, doxycycline, minocycline, and sarecycline) in the modulation of human mesenchymal stem cells’ activity revealed their potential to enhance osteogenic differentiation, as evidenced by the modulation of the actin cytoskeleton, increased osteogenic gene expression, and ALP activity. The present findings indicate that TETRA and DOXY exert their effects through increased Wnt signaling, while MINO and SARE may mediate their effects through an alternative mechanism, potentially involving increased Hedgehog signaling. It is noteworthy that MINO and SARE exhibited the highest levels of osteogenic induction, comparable to those achieved with the gold standard in vitro osteogenic induction using dexamethasone, ascorbic acid, and β-glycerophosphate. These outcomes could be intricately linked to variances in the chemical structure, particularly within the C7 position of the D ring, thus possibly underpinning the achieved outcomes.

The potential of TCs to promote osteogenic differentiation holds promise for targeted treatments based on specific bone-related disorders. Further investigation into the precise signaling pathways and mechanisms involved will undoubtedly contribute to the development of novel and effective regenerative therapies.

## Figures and Tables

**Table 1:** The chemical structure, characteristics, and source of the assessed TCs.

	Tetracycline-HCl	Doxycycline-HCl	Minocycline-HCl	Sarecycline-HCl
Chemical Structure	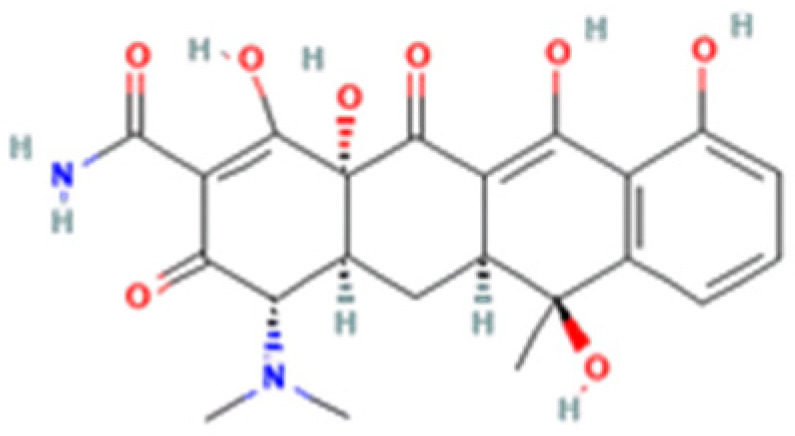	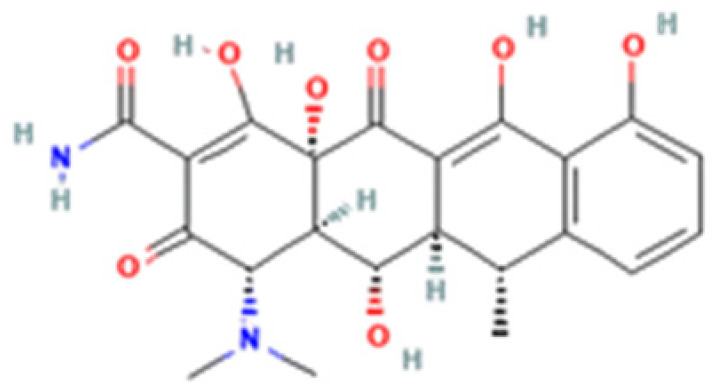	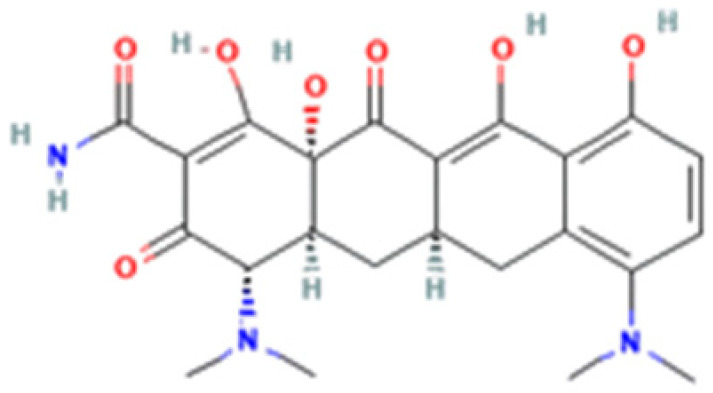	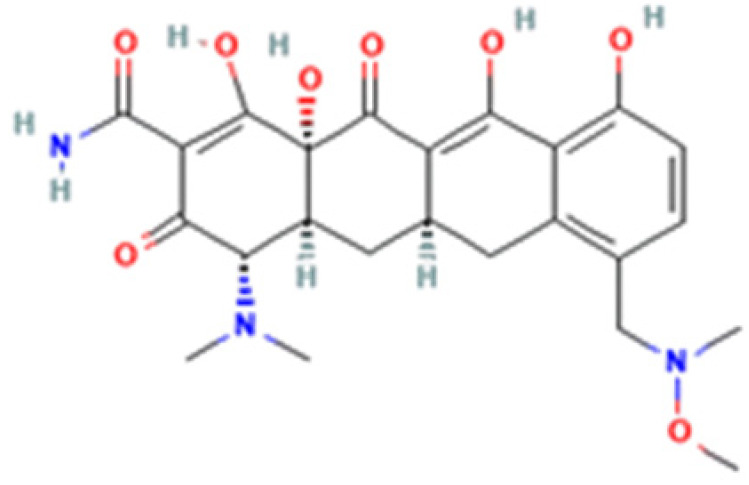
Generation	1st Generation	2nd Generation		3rd Generation
Modification (to TC)	___	C5 (R4) addition of OH C6 (R2) removal of OH	C7 (R1) addition of N(CH_3_)_2_ C6 (R2 and R3) removal of OH and CH_3_	C7 (R1) addition of methoxy-methyl-amino-methyl group C6 (R2 and R3) removal of OH and CH_3_
Obtained from/Batch	Sigma-Aldrich/T8032	Sigma-Aldrich/D3447	Sigma-Aldrich/M9511	Adooq Bioscience/P005672

From National Center for Biotechnology Information (2023). PubChem Compound Summary for CID 54675776 (tetracycline), CID 54671203 (doxycycline), CID 54675783 (minocycline) and CID 54681908 (sarecycline). Accessed on 9 September 2023, https://pubchem.ncbi.nlm.nih.gov.

**Table 2:** The unique assay ID (Bio-Rad, Hercules, CA, USA) of the used primers.

Gene	Assay ID	Gene	Assay ID
*ACTB*	qHsaCED0036269	*PTCH1*	qHsaCEP0055042
*RUNX2*	qHsaCED0044067	*GLI2*	qHsaCEP0057630
*COL1A1*	qHsaCED0043248	*HEY1*	qHsaCED0046240
*BGLAP*	qHsaCED0038437	*HES1*	qHsaCED0006922
*SP7*	qHsaCED0003759	*AXIN2*	qHsaCID0017930
*SPARC*	qHsaCID0010332	*TWIST1*	qHsaCED0003856

Abbreviations: *ACTB*: actin-beta; *AXIN2*: axis inhibition protein 2; *BGLAP*: osteocalcin; *COL1A1*: Collagen Type I Alpha 1; *GLI2*: Glioma-Associated Oncogene Family Zinc Finger 2; *HES1*: Hes Family BHLH Transcription Factor 1; *HEY1*: Hes-Related Family BHLH Transcription Factor With YRPW Motif 1; *PTCH1*: Protein-Patched Homolog 1; *RUNX2*: Runt-related transcription factor 2; *SP7*: osterix; *SPARC*: osteonectin; *TWIST1*: Twist Family BHLH Transcription Factor 1.

## Data Availability

The data presented in this study are available on request from the corresponding author.
